# Inter-Individual Variability in Postural Control During External Center of Mass Stabilization

**DOI:** 10.3389/fphys.2021.722732

**Published:** 2022-01-03

**Authors:** Daša Gorjan, Nejc Šarabon, Jan Babič

**Affiliations:** ^1^Department of Automation, Biocybernetics and Robotics, Laboratory for Neuromechanics and Biorobotics, Jožef Stefan Institute, Ljubljana, Slovenia; ^2^Faculty of Health Sciences and Andrej Marušicˇ Institute, University of Primorska, Koper, Slovenia; ^3^InnoRenew CoE, Izola, Slovenia; ^4^S2P, Science to Practice, d.o.o., Laboratory for Motor Control and Motor Behavior, Ljubljana, Slovenia; ^5^Faculty of Electrical Engineering, University of Ljubljana, Ljubljana, Slovenia

**Keywords:** postural control, inverted pendulum, external stabilization, hierarchical clustering, postural variability

## Abstract

Understanding the relation between the motion of the center of mass (COM) and the center of pressure (COP) is important to understand the underlying mechanisms of maintaining body equilibrium. One way to investigate this is to stabilize COM by fixing the joints of the human and looking at the corresponding COP reactions. However, this approach constrains the natural motion of the human. To avoid this shortcoming, we stabilized COM without constraining the joint movements by using an external stabilization method based on inverted cart-pendulum system. Interestingly, this method only stabilized COM of a subgroup of participants and had a destabilizing effect for others which implies significant variability in inter-individual postural control. The aim of this work was to investigate the underlying causes of inter-individual variability by studying the postural parameters of quiet standing before the external stabilization. Eighteen volunteers took part in the experiment where they were standing on an actuated cart for 335 s. In the middle of this period we stabilized their COM in anteroposterior direction for 105 s. To stabilize the COM, we controlled the position of the cart using a double proportional–integral–derivative controller. We recorded COM position throughout the experiment, calculated its velocity, amplitude, and frequency during the quiet standing before the stabilization, and used these parameters as features in hierarchical clustering method. Clustering solution revealed that postural parameters of quiet standing before the stabilization cannot explain the inter-individual variability of postural responses during the external COM stabilization. COM was successfully stabilized for a group of participants but had a destabilizing effect on the others, showing a variability in individual postural control which cannot be explained by postural parameters of quiet-stance.

## Introduction

Maintaining postural equilibrium is fundamental for standing upright. This is achieved by coordinating motor commands and responses based on multiple sensory inputs and biomechanical constraints (Nashner, 1997). Measures of body sway as a movement of center of mass (COM) or center of pressure (COP) are commonly used to evaluate the performance of standing posture (Palmieri et al., 2002).

Investigating underlying mechanisms of postural control requires understanding of the relationship between COM and COP. Traditionally, COP variations are assumed to correct the unstable COM position back to the equilibrium (Johansson et al., 1988; Peterka, 2000). In contrast to traditional theories, other studies found that an additional purpose of the COP oscillations is to increase the sensory information flow from the environment (van Emmerik and van Wegen, 2002; Mochizuki et al., 2006; Stergiou et al., 2006; Carpenter et al., 2010).

One way to investigate the link between COM and COP is to stabilize the COM and observe parameters of COP oscillations, which was already done by Carpenter et al. (2010). However, their approach to COM stabilization was based on constraining the participant’s joint movement by bracing them to a fixed board which did not allow for an ecological standing posture. Although this makes human body mechanically comparable to the inverted pendulum (Winter et al., 1998; Chagdes et al., 2013), it substantially affects postural control (Gage et al., 2004). Based on this, Gorjan et al. (2021) developed a method for stabilization of COM without mechanically constraining the natural movement of subjects. The method is based on a pulling system attached around the hips that stabilizes the motion of the COM by applying feedback forces. Even though this method does not constrain the joint motion of the subjects, it does have a mechanical effect by applying forces on the human body and this could have an effect on postural control.

To fully avoid constraining the body during the COM stabilization, we designed a novel methodological approach to stabilize the COM by moving the cart on which the participants can stand. This way, no mechanical forces are applied at the human body, except through the ground reaction forces. The cart stabilization method was designed based on the inverted pendulum model and the fact that the inverted pendulum can be stabilized by putting it on a moving cart (Lozano et al., 2000; Muskinja and Tovornik, 2006). Preliminary experiments using this stabilization method showed that COM of only a subgroup of participants was stabilized while it had a destabilizing effect for the others. Since the movement of the cart was controlled based on the movement of COM, the participant’s reactions to the stabilization cannot be investigated by classical postural analysis based on COM motion. Nevertheless, according to Hsiao-Wecksler et al. (2003), it is possible to predict an individual’s dynamic response to a mild perturbation only by analyzing quiet-stance data.

The aim of this study was to perform an experiment where a group of participants is subjected to external COM stabilization and to examine the possible relationship between the postural parameters of quiet standing and the susceptibility of an individual to external COM stabilization. We hypothesized that there should be a specific pattern of postural, kinematic and frequential parameters of COM during quiet standing associated with the individuals that are stabilized and those who are destabilized by the external COM stabilization.

## Materials and Methods

### Participants

Eighteen healthy young adults (seven females) participated in the study. Their average age was 23.2 years (*SD* = 2.1 years), height 174.7 cm (*SD* = 11.1 cm), and body mass 70.2 kg (*SD* = 12.8 kg). The participants’ individual characteristics are presented in Table 1. The age (18 to 30 years) was the inclusion criteria, and the exclusion criteria were history of neurological or musculoskeletal disorders or recent injury. The participants gave their written informed consent before participating in this study which was approved by the National Medical Ethics Committee of the Republic of Slovenia (No. 339/2017/7). Participants were divided into two groups based on the effect that stabilization method had on them. Seven participants were stabilized by the stabilization system and on the remaining 11 it had a destabilization effect in form of losing their balance (large oscillations or movement of the cart).

**Table 1:** Characteristics of participants with subject numbers (m = male, f = female).

Subject number	1	2	3	4	5	6	7	8	9	10	11	12	13	14	15	16	17	18
Gender	f	m	f	m	m	m	f	m	m	f	f	m	m	f	m	m	m	f
Age	28	26	25	22	26	22	26	23	23	20	24	22	22	22	24	21	25	24
Height (cm)	165	184	155	184	172	186	167	180	182	158	165	191	177	160	175	183	190	172
Weight (kg)	65	76	51	90	77	69	58	85	74	54	59	94	67	58	60	82	82	63

### Study Design

**Figure 1:**
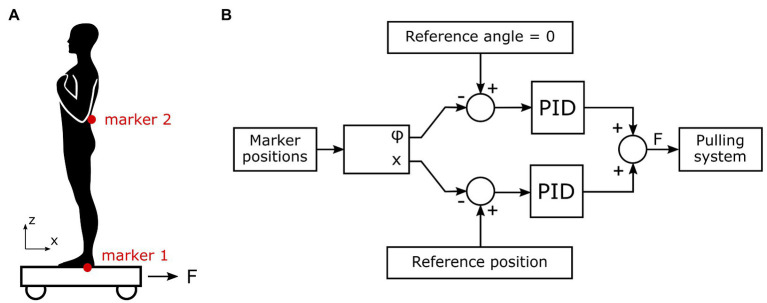
**(A)** Human participant standing on the cart and the location of the motion capture markers. Marker 1 is placed on the cart below the ankle joint and marker 2 is placed on the L5 vertebra as an approximation of the center of mass (COM) position. **(B)** Scheme of cart controller to stabilize the participant’s COM. *φ* is the angle between vertical line and the line that connects both markers, x is the horizontal position of marker 1, and F is the resulting force applied on the cart.

The setup consisted of a cart with an integrated force plate (Kistler Instrumente AG, Winterthur, Switzerland) on which the participants stood. The cart was mounted on the rails that constrained the motion of the cart in anteroposterior direction. The cart was actuated by two motors located at both sides of the rail that pulled the cart with a steel wire. Location of the participant’s COM was approximated by an infra-red marker placed under the L5 vertebra and recorded in real time by Optotrak motion capture system (3D Investigator, Northern Digital Inc., Waterloo, Canada) with 1,000 Hz sampling frequency. The motors were controlled by a double proportional–integral–derivative (PID) controller based on the location of the participant’s COM. In effect, our setup allowed us to arbitrarily move the participant’s COM in anteroposterior direction (Figure 1). We set the parameters of the PID controller by first rough-tuning them to stabilize an aluminum inverted pendulum with a single rotational joint at the bottom and then fine-tuning them for the size and weight of the human body. The parameters were the same for all participants.

Participants were instructed to quietly stand on the cart with their feet hip-width apart. To exclude the effects of arm motion on quiet standing, we asked the participants to hold their arms crossed over the chest (Milosevic, et al., 2011). During the first 115 s, the cart was fixed and did not move, then it stabilized the participant’s COM for 105 s and went back to the fixed mode for another 115 s. To avoid possible anticipatory behavior, we did not educate the participants about the time when the cart will perform the stabilization. In total, the participants were standing on the cart for 335 s.

### Data Processing

To remove the noise, we first filtered the COM data obtained by the motion capture system using the second-order Butterworth low-pass filter with a 5 Hz cutoff frequency. We then calculated the RMS amplitude of COM and the RMS velocity of COM. Further, we calculated the power spectral density with fast Fourier transformation. Finally, we divided the spectrum to low frequency range (LF: 0.02–0.1 Hz), medium frequency range (MF: 0.1–1 Hz), and high frequency range (HF: 1–10 Hz) and calculated the area under the curve (AUC) for each range (Fujimoto et al. 2014).

### Clustering and Statistical Analysis

There are several techniques available to investigate the correlations between different biomechanical parameters and to understand the movement variability of human subjects. Using supervised methods, correlations between the group of subjects can be performed using discriminant analysis paradigm (Tibarewala and Ganguli, 1982) where the groups of subjects need to be predefined. Moreover, several unsupervised methods were proven suitable to get insight into gait analysis (Holzreiter and Köhle, 1993; Begg et al., 2005; Horst et al., 2019). However, most of this research was predominantly oriented into classification of biomechanical parameters into predefined subject groups, ignoring the problem of unbiased discovering the underlying biomechanical parameters that lead to the changes of movement variability. Powerful methods to investigate inter-individual variability in human motion patterns without manual predefinition of groups are the clustering techniques. They were used to investigate variability in complex movements, such as walking and running (Mulroy et al., 2003; Bartlett et al., 2014). Moreover, hierarchical clustering algorithms have been used for mining gait patterns based on stride length and step frequency (Xu et al., 2006) and to investigate universal and individual characteristics of postural sway during quiet standing (Yamamoto et al., 2015). Multivariate clustering techniques were used for discovering human balance patterns and finding the association between COP parameters and different demographic and health characteristics of the participants (Malik and Lai, 2017).

Based on these previous methodological approaches, we used Ward hierarchical clustering technique which selects a pair of clusters to merge them at each step based on minimal error sum of squares (Mojena, 1977). The cluster solutions were generated using the anteroposterior COM displacement data from the 115 s period of quiet standing before the cart stabilized the participants. We selected RMS amplitude of COM, RMS velocity of COM, and AUC of HF COM motion as features for clustering, since postural, frequential, and kinematic parameters together thoroughly describe the movement of the human (Stins et al., 2011; Luca, 2016). We used *Z*-score standardization method to have equal influence of all included parameters (Mohamad and Usman, 2013).

To investigate possible effects of age, height, and weight of the participants on the stabilization and on the categorization results of the clustering, we compared the means of these parameters using Welch t-test. All statistical analyses were performed using R, version 4.0.2.

## Results

**Figure 2:**
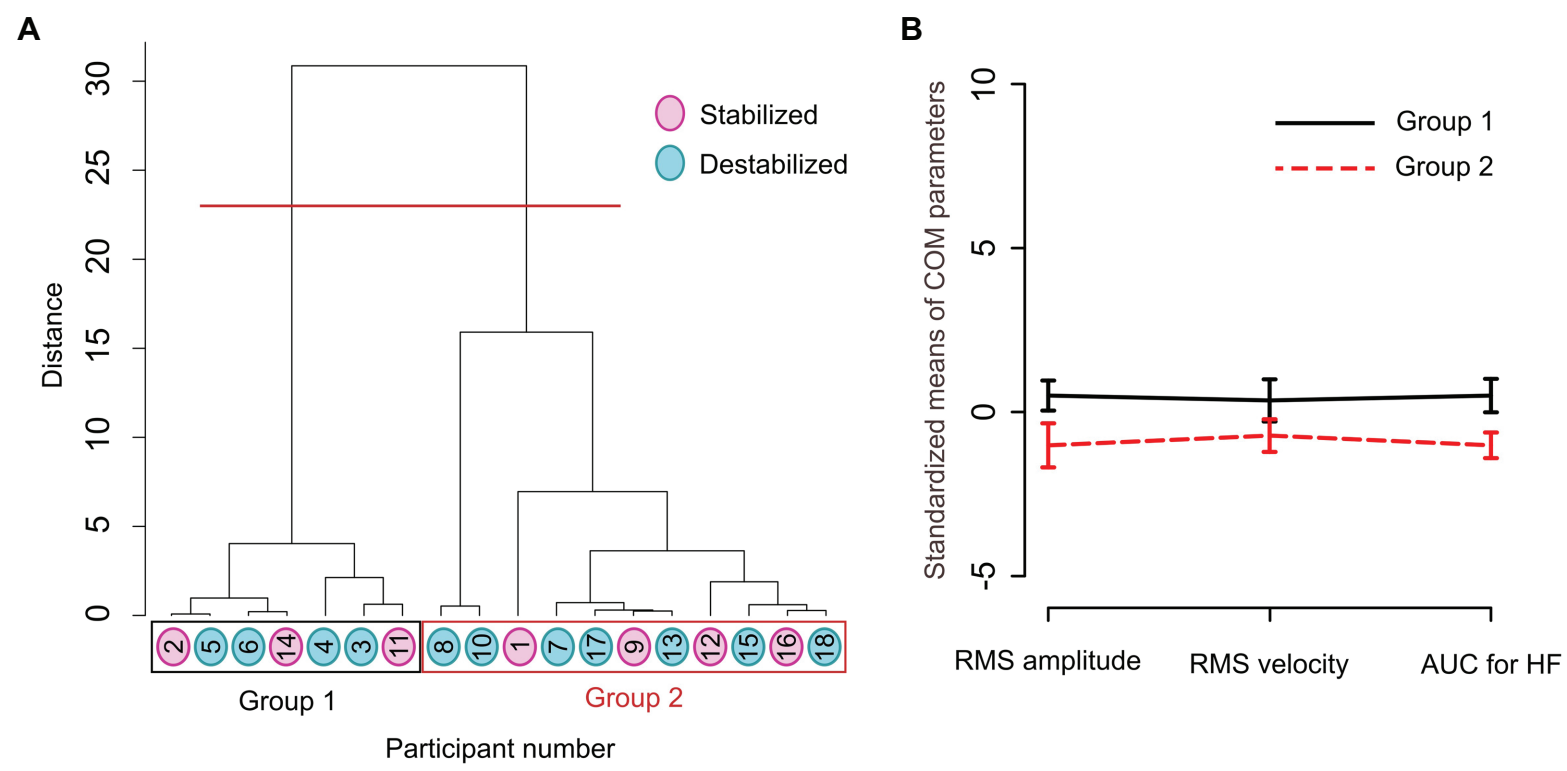
**(A)** Dendrogram of the process of merging the units into groups with Ward’s hierarchical clustering method based on the amplitude, velocity, and frequency parameter of COM as features. Our clustering solution separated participants into 2 groups. Participants stabilized by the cart stabilization method are marked with pink and the destabilized participants are marked in blue. **(B)** Means and standard deviations of standardized (subtracted mean and multiplied by 1,000) COM parameters for two groups grouped by Ward’s hierarchical clustering method.

Participants stabilized by our stabilization method were 1, 2, 9, 11, 12, 14, 16 (pink circles on Figure 2A) and the ones who were destabilized were 3, 4, 5, 6, 7, 8, 10, 15, 17, 18 (blue circles on Figure 2A). On the other hand, clustering based on the RMS amplitude of COM, RMS velocity of COM, and AUC of HF COM motion separated the participants into Group 1 with participants 2, 3, 4, 5, 6, 11, 14 and into Group 2 with participants 1, 7, 8, 9, 10, 12, 13, 15, 16, 17, 18. This indicates that the clustering analysis based on the selected parameters of quiet standing did not separate the participants into those that were stabilized by our method and those that were destabilized. Oppositely, the clustering analysis separated participants into two groups regardless of stabilization or destabilization effect of our method on them.

Moreover, RMS amplitude, RMS velocity, and AUC of HF COM motion of the participants grouped in Group 1 are higher compared to the participants grouped in Group 2 (Figure 2B). The two groups are different in terms of included parameters, but that difference does not explain the response of participants to the external stabilization.

**Figure 3:**
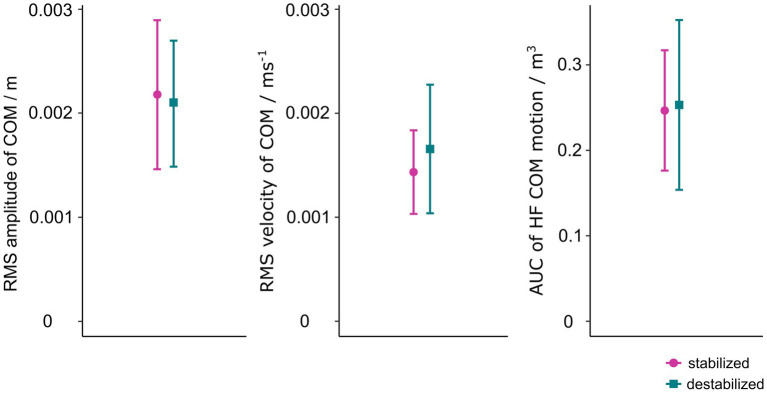
Means and standard deviations of COM parameters of quiet standing for participants that were stabilized by cart stabilization method and for those that were not.

Means and standard deviations of the clustering features for participants that were stabilized by our stabilization method and for those that were destabilized are presented on Figure 3.

Neither the participants’ age, their height, nor their weight had an effect neither on stabilization nor on the categorization of participants using the clustering method. The statistical results of all relevant comparisons are presented in Table 2.

**Table 2:** Statistical results of comparing participants’ age, height, and weight based on stabilization and clustering categorization.

	Stabilised vs. Distabilised			Group 1 vs. Group 2		
	*t*	*df*	*p*	*t*	*df*	*p*
Age	−0.15	10.46	0.88	0.41	14.86	0.69
Height	−0.27	12.06	0.79	−0.71	10.95	0.49
Weight	−0.61	13.01	0.55	−0.42	12.47	0.68

## Discussion

The cart stabilization method stabilized 7 out of 18 participants and had a destabilizing effect for the rest. Even though the method was the same for all participants, their response to the stabilization was different. Our results show that neither amplitude, velocity, nor the frequency parameters of COM during quiet standing cannot explain the inter-individual variability of postural responses during the external COM stabilization.

Clustering techniques were already used for analyzing postural data in similar studies (Xu et al., 2006; Malik and Lai, 2017). We selected COM frequency, amplitude, and velocity as features for the clustering algorithm, since these three parameters thoroughly describe the quiet standing movement. However, we also explored other subgroups of measured parameters (knee/hip/ankle joint angles, COM/COP velocity, amplitude, frequency), but no better clustering solutions were obtained in terms of equality of cluster sizes and according to the Ward criterion function (analyses not included in this paper). There is still room for further investigation of parameters that would better explain variability of postural sway. For example use of nonlinear parameters could improve the characterization of sway dynamics (Sabatini, 2000; Ghomashchi et al., 2011).

A viable possibility would be that the effect of stabilization could be explained by the differences of participants’ age, height, or weight. The effects of anthropometric characteristics on standing balance were previously studied (Kejonen, et al., 2003; Alonso, et al., 2015). They found a small effect of anthropometric characteristics on balance and that the height has the most influence on it. Even though the effects of anthropometry on balance are small, it could be enough to explain the differences of the COM stabilization used in our study. However, neither the age, height, nor the weight had an effect neither on stabilization nor on the categorization of the participants. We can therefore conclude that, in our case, these parameters were not determinant of the stabilization effects. We can also conclude that neither the age, height, nor the weight had an effect on the differences of COM amplitude, velocity, and frequency parameters found between Group 1 and Group 2. Nevertheless, there are several other biomechanical parameters (e.g., strength, physical ability levels, and ankle mobility) that affect postural control and whose possible effects on external COM stabilization should be explored in the future studies.

An important question is whether we can investigate responses to the cart stabilization method based on the quiet-stance data. Hsiao-Wecksler et al. (2003) found that it is possible to predict dynamic response of individuals to a mild perturbation only by analyzing quiet-stance data. Nevertheless, we could not predict the responses to stabilization based on the quiet-stance data, possibly because Hsiao-Wecksler et al. (2003) used a discrete impulse disturbance applied as a pull to the waist while we used a continuous, movement-dependent COM stabilization.

The analysis of the immediate postural reaction on the stabilization method has the potential to explain the inter-individual postural control variability (Moore et al., 1988). However, the nature of the cart stabilization method where COM was driving the cart movement, prevents us to separate the cause and the consequence of the COM movement. To analyze the immediate reaction to the stabilization, an additional experiment with the same participants investigating reaction to the discrete perturbation should be carried out. Moreover, the effect of the subjects knowing when the stabilization is initiated might have an important effect on their postural responses. This would allow us to elucidate if a possible difference in immediate reaction to the perturbation correlates with the response to the external stabilization.

Another possible cause for the different responses could be the differences in the sensitivity to the threat (Johnson et al., 2019). Since the participants were not informed about the stabilization, they could consider it as a postural threat. To investigate the difference in perception of postural threat, an additional experiment should be held where participants would be exposed to different levels of postural threat while we would measure the electro-dermal activity, which would allow us to compare the level of stress caused by the threat (Sibley et al., 2009, 2010).

There are many different algorithms for the stabilization of the inverted pendulum (Lozano et al., 2000; Muskinja and Tovornik, 2006) and we used one of the simplest version based on a pair of PID controllers with fixed parameters for all participants. It is important to note that the individual responses to stabilization did not correlate neither with the height nor with the weight of the participants. Furthermore, individualized tuning of the PID parameters would bias the experiment since the participants would have to be involved in the tuning procedure and would hence experience the external stabilization before the actual experiment.

Different responses to the external COM stabilization imply inter-individual variability in postural control. Even though general principles of postural control have been studied for decades, differences between individuals still cannot be fully explained (Foisy and Kapoula, 2016; Coste et al., 2021). For instance, it is widely accepted that the postural sway increases when eliminating the visual sensory information; however, there is a large group of people who sway less with their eyes closed (Lacour et al., 1997; Chiari et al., 2000). In conclusion, our study shows that the variability in individual postural control cannot be explained by postural parameters of quiet-stance. Further experiments are needed to understand the roots of postural variability and, among others, suggest how to improve the stabilization method to be applicable for a larger population.

## Data Availability Statement

The raw data supporting the conclusions of this article will be made available by the authors, without undue reservation.

## Ethics Statement

The studies involving human participants were reviewed and approved by National Medical Ethics Committee of the Republic of Slovenia (No. 339/2017/7). The patients/participants provided their written informed consent to participate in this study.

## Author Contributions

All authors have made substantial contributions to the conception and design of the study, acquisition of data, analysis, interpretation of data, drafting the manuscript and revising it critically for important intellectual content.

## Funding

This work was supported by the European Union’s Horizon 2020 through the AnDy project (contract nr. 731540) and by the Slovenian Research Agency (research core funding nr. P2-0076).

## Conflict of Interest

The authors declare that the research was conducted in the absence of any commercial or financial relationships that could be construed as a potential conflict of interest.

## Publisher’s Note

All claims expressed in this article are solely those of the authors and do not necessarily represent those of their affiliated organizations, or those of the publisher, the editors and the reviewers. Any product that may be evaluated in this article, or claim that may be made by its manufacturer, is not guaranteed or endorsed by the publisher.
